# Motoric Cognitive Risk and Incident Dementia in Older Adults

**DOI:** 10.1001/jamanetworkopen.2023.38534

**Published:** 2023-10-19

**Authors:** Jeehae Chung, Seonjeong Byun

**Affiliations:** 1 Industry-Academic Cooperation Foundation, The Catholic University of Korea, Seoul, Korea; 2 Department of Neuropsychiatry, Uijeongbu St Mary’s Hospital, College of Medicine, The Catholic University of Korea, Seoul, Korea

## Abstract

**Importance:**

Motoric cognitive risk (MCR) is a novel predementia syndrome; however, whether it can estimate dementia in a nationwide population or has additive estimation validity over cognitive or motoric components alone remains unknown.

**Objective:**

To examine whether modified MCR, which incorporates the timed-up-and-go and one-leg-standing tests, improves estimation validity for incident dementia over using cognitive or motoric components alone.

**Design, Setting, and Participants:**

This nationwide cohort study evaluated data from individuals aged 66 years who participated in the National Screening Program for Transitional Ages in Korea from January 1, 2009, to December 31, 2013, and examined the association between MCR and incident dementia using Cox proportional hazards regression analysis. Data were collected from the index date (the date on which the participant had the screening) until dementia onset, death, or the end of the follow-up period, whichever came first. The 2 subtypes were defined as subjective cognitive declines with timed-up-and-go impairment or one-leg-standing impairment. The data set was generated with permission from the Korean National Health Insurance Service, and data analysis was conducted from August 2, 2021, to January 31, 2022. Individuals diagnosed with dementia or psychotic disorders or those who had a documented history of dementia medication use before the index date were excluded.

**Main Outcomes and Measures:**

The main outcome was incidence of dementia, defined as an individual receiving their first dementia medication with the relevant *International Statistical Classification of Diseases and Related Health Problems, Tenth Revision*, codes after the index date.

**Results:**

Among the 1 137 530 participants (53.7% women), 15 380 (1.4%) met the MCR criteria for the timed-up-and-go subtype, and 32 910 (2.9%) met the criteria for the one-leg-standing subtype. The mean (SD) follow-up period was 7.02 (1.38) years. Participants with MCR demonstrated an approximately 2-fold higher risk of incident dementia than those without MCR (timed-up-and-go subtype, adjusted hazard ratio, 2.03; 95% CI, 1.94-2.13; one-leg-standing subtype, adjusted hazard ratio, 2.05; 95% CI, 1.98-2.12).

**Conclusions and Relevance:**

In this cohort study of participants aged 66 years of the National Screening Program for Transitional Ages, modified motoric cognitive risk had higher adjusted hazard ratios of incident dementia than individual cognitive or motoric components. Motoric cognitive risk may be a practical screening tool for estimating dementia among individuals in their mid-60s ; however, further investigation of the clinical and neurobiological aspects is necessary.

## Key Points

**Question:**

Is modified motoric cognitive risk (MCR) that incorporates the timed-up-and-go (TUG) and one-leg-standing (OLS) tests able to estimate the risk of incident dementia?

**Findings:**

In this large, nationwide cohort study with 1 137 530 individuals aged 66 years, both the MCR-TUG and MCR-OLS groups exhibited an approximately 2-fold significantly higher risk of incident dementia than the non-MCR groups. The MCR group exhibited a higher cumulative incidence of dementia and significantly shorter follow-up period than those in non-MCR groups.

**Meaning:**

The findings of this study suggest that modified MCR may be used as a practical screening tool for estimating dementia among individuals in their mid-60s.

## Teaser

This cohort study examines the use of motoric cognitive risk testing to screen for risk of incident dementia in individuals aged 66 years in South Korea.

## Introduction

Dementia is a major global health problem that affects 55 million people worldwide with an estimated economic burden of 1.1% of the global gross domestic product that is forecast to double by 2030.^1,2^ The impact of dementia on public health and the absence of a cure underscore the need for early detection to prevent and/or delay its onset. Emerging evidence indicates that gait or balance changes may be viable biomarkers for the early identification of individuals at high risk for dementia.

Slow gait has been associated with accelerated cognitive decline and the risk of mild cognitive impairment and dementia development, particularly vascular dementia (VD) and other non–Alzheimer disease (AD) dementias.^3,4,5^ Motoric cognitive risk (MCR) is defined as a novel predementia syndrome characterized by the presence of subjective cognitive declines (SCDs) and a slow gait.^6^ Motoric cognitive risk has an independent estimation value distinct from that of mild cognitive impairment.^7^ Most MCR studies have used slow walking speed as a motoric component; however, MCR subtypes have been defined using gait parameters other than speed and have been shown to have varying cognitive profiles and risk factors.^8,9^ The timed-up-and-go (TUG) test is a reliable and valid tool for easily quantifying motor function in older adults in a clinical setting by examining lower extremity function and gait speed.^10^ Decreased TUG performance is associated with a higher risk of cognitive decline, dementia, and all-cause mortality among community-dwelling older adults.^11,12^ The one-leg-standing (OLS) test assesses postural balance and is used in daily health care practice. Decreased OLS performance is a marker of advanced dementia and cognitive decline.^13^ Therefore, we hypothesized that a modified MCR using TUG and OLS tests as motoric parameters could estimate incident dementia.

Nationwide population-based studies reporting that impaired TUG and SCD estimate dementia are limited.^11,12,14^ Moreover, to our knowledge, no studies have examined whether MCR-TUG estimates dementia in a large, nationwide, community-based population, whether it has additive estimation validity over using TUG or SCDs alone, or whether OLS or MCR-OLS can estimate dementia.

This nationwide cohort study aimed to examine the risk of incident dementia in participants with and without MCR. We evaluated whether modified MCR, incorporating MCR-TUG and MCR-OLS, has additive estimation validity for incident dementia over using cognitive or motoric components alone.

## Methods

### Data Sources and Study Population

Data were obtained retrospectively from the Korean National Health Insurance Service database (eMethods in Supplement 1). We analyzed data from participants in the 66-year-old National Screening Program for Transitional Ages (NSPTA)^15^ between January 1, 2009, and December 31, 2013. This study was approved by the institutional review board of The Catholic University of Korea Uijeongbu St Mary’s Hospital and adhered to the Strengthening the Reporting of Observational Studies in Epidemiology (STROBE) reporting guideline for cohort studies. The requirement for informed consent was waived because of data anonymity.

### Eligibility Criteria

We included participants of the 66-year-old NSPTA from 2009 to 2013 with values for primary independent variables: TUG, OLS, and prescreening Korean Dementia Screening Questionnaire (KDSQ-P).^16^ Exclusion criteria were individuals diagnosed according to the *International Statistical Classification of Diseases and Related Health Problems, Tenth Revision*, with dementia (*ICD-10* codes F00-03, F051, F067, and G30-31) or psychotic disorder (*ICD-10* code F20-29) before the index date (the date the participant underwent NSPTA); had a documented history of dementia medication use (donepezil, rivastigmine, galantamine, or memantine) before the index date; or had missing or duplicate primary independent variable data.

### Study Variables

#### Outcome Variable

The incidence of dementia was defined as any individual receiving the first dementia medication prescription and *ICD-10* codes F00-03, F051, F067, or G30-31 post–index date according to the Korean National Health Insurance Service reimbursement system. The dementia subtypes AD (*ICD-10* code F00 or G30) and VD (*ICD-10* code F01) were also considered. While defining dementia based solely on *ICD-10* codes risks overestimation,^17^ adding medication prescription enhances reliability since clinicians are required to document both the dementia *ICD-10* codes and neuropsychological test results to prescribe cognitive-enhancing medications.

#### Independent Variables

The TUG and OLS tests were conducted as part of the NSPTA. The TUG test recorded the time taken by participants to stand up from a chair, walk 3 m, turn around, walk back to the chair, and sit down again.^15^ The participants were allowed to wear shoes or use walking aids, if necessary. Impaired TUG was defined as a test duration greater than 1 SD from the sex-specific means, with a cutoff of 12 seconds for men and 13 seconds for women. The OLS test recorded the time taken by participants standing on one leg with their eyes open or closed to lose balance or touch the floor with their non–weight-bearing leg. Impaired OLS was defined as a test duration less than 1 SD from the sex- and test condition–specific means (eTable 1 in Supplement 1).

The KDSQ-P is a self-reported questionnaire consisting of 5 questions with higher scores (range, 0-10) indicating higher SCD. Participants who scored 4 or higher^16^ were defined as the SCD group (eMethods in Supplement 1).

Motoric cognitive risk is defined as the coexistence of SCD and an objective decline in motor function.^6^ We classified modified MCR into 2 subtypes: MCR-TUG, denoting participants with SCD and impaired TUG, and MCR-OLS, denoting participants with SCD and impaired OLS.

### Covariates

Sex and income were included as covariates. Income was divided into 20 levels, which we recategorized into 4 groups: group 1 (ventiles 1-8 [lowest levels]), group 2 (ventiles 9-15), group 3 (ventiles 16-20), and group 4 (Medicaid beneficiaries). We controlled for lifestyle factors such as physical activity, smoking, and alcohol consumption. Clinical factors assessed included body mass index; blood pressure; fasting blood glucose, hemoglobin, high-density cholesterol, low-density cholesterol, and triglyceride levels, hearing, and depressive symptoms. Depressive symptoms were self-reported using 3 questions extracted from the Short-Form Geriatric Depression Scale,^18^ with higher scores (0-3) indicating more depressive symptoms. Medical comorbidities were defined as a diagnosis of hypertension, diabetes, dyslipidemia, heart disease, or stroke and a corresponding medication prescription. For missing covariate values, the chained equations (MICE) algorithm, which generated multiple imputed data sets and estimated missing values based on the correlations between variables,^19^ was used for multivariate imputation.

### Statistical Analysis

Data analysis was conducted between August 2, 2021, and January 31, 2022. Differences between the MCR and non-MCR groups (MCR-TUG vs non–MCR-TUG and MCR-OLS vs non–MCR-OLS) were determined using a 2-sided, independent *t* test and χ^2^ test. Cox proportional hazards regression analysis was used to evaluate the influence of modified MCR on incident dementia. The follow-up period was from the index date to the date of dementia onset, death, or the end of the follow-up period (December 31, 2018), whichever came first. The adjusted hazard ratios (AHRs) for impaired TUG, impaired OLS, SCD, MCR-TUG, and MCR-OLS in estimating incident dementia while controlling for covariates were initially analyzed using an unadjusted model. Then, covariate adjustment was performed using 4 additional models: model 1 controlled for sex and income, model 2 included lifestyle factors (physical activity, smoking, and alcohol consumption), model 3 included clinical factors (body mass index; blood pressure; fasting blood glucose, hemoglobin, high-density lipoprotein cholesterol, low-density lipoprotein cholesterol, and triglyceride levels; hearing; and depressive symptoms), and model 4 included medical comorbidities (hypertension, diabetes, dyslipidemia, heart disease, and stroke).

Propensity score matching was applied to the MCR-TUG and MCR-OLS groups; the impaired TUG, impaired OLS, and SCD groups; and their respective reference groups. Matching (1:1) was conducted for all variables designated as covariates to minimize influence-confounding variables.^20,21^

Sensitivity analysis was conducted to confirm the robustness of our findings. Participants diagnosed with dementia within 1 year of the index date were excluded to focus on incident dementia cases (sensitivity analysis I). The TUG and OLS outliers were excluded using the semi-IQR method, considering the distribution of the data (sensitivity analysis II).^22^

We examined whether impaired TUG, impaired OLS, SCD, MCR-TUG, and MCR-OLS associated with the incidence of dementia varied depending on the subtype (AD or VD). Statistical analysis was conducted using 2-tailed tests at *P* < .05, and 95% CIs. SAS Enterprise Guide, version 9.4 (SAS Institute Inc) and R Studio, version 4.0.3 (R Foundation for Statistical Computing) were used (Survival, version 3.2-7; Survminer, version 0.4.6; and MICE, version 3.7.0).

## Results

A total of 1 245 831 participants underwent NSPTA from January 1, 2009, to December 31, 2013. Among these, we excluded 60 478 owing to missing or duplicate primary variable data, 41 319 diagnosed with dementia, and 6504 diagnosed with psychotic disorders before the index date. Following these exclusions, 1 137 530 participants representing approximately 60% of the Korean population aged 66 years between 2009 and 2013 were included (eFigure in Supplement 1).^23^

### Participant Characteristics and MCR

The participants comprised 526 902 men (46.3%) and 610 628 women (53.7%), and 96.7% were Korean National Health Insurance subscribers. Most participants engaged in 1 or more physical activities per week (74.0%), were never smokers (69.4%), and consumed fewer than 7 units of alcohol per week (84.4%) (Table 1). Missing covariate values imputed constituted approximately 0% to 2.4% for each variable.

**Table 1.** Descriptive Characteristics of the Participants in the MCR and Non-MCR Groups

Variable	Total, No. (%) (N = 1 137 530)	MCR-TUG			MCR-OLS		
		MCR, No. (%) (n = 15 380)	Non-MCR, No. (%) (n = 1 122 150)	Difference^a^	MCR, No. (%) (n = 32 910)	Non-MCR, No. (%) (n = 1 104 620)	Difference^a^
Sex							
Male	526 902 (46.3)	6268 (40.8)	520 634 (46.4)	194.2^b^	12 567 (38.2)	514 335 (46.6)	899.0^b^
Female	610 628 (53.7)	9112 (59.2)	601 516 (53.6)		20 343 (61.8)	590 285 (53.4)	
Income, ventile							
1-8 (Lowest)	320 102 (28.1)	3768 (24.5)	316 334 (28.2)	1620.9^b^	8060 (24.5)	312 042 (28.3)	2560.9^b^
9-15	367 081 (32.3)	4989 (32.4)	362 092 (32.3)		10 699 (32.5)	356 382 (32.3)	
16-20	412 922 (36.3)	5245 (34.1)	407 677 (36.3)		11 492 (34.9)	401 430 (36.3)	
Medicaid	37 425 (3.3)	1378 (9.0)	36 047 (3.2)		2659 (8.1)	34 766 (3.2)	
Lifestyle factors							
Physical activity							
Never	295 818 (26.0)	5112 (33.2)	290 706 (25.9)	432.1^b^	10 509 (31.9)	285 309 (25.8)	609.6^b^
≥Once a wk	841 712 (74.0)	10 268 (66.8)	831 444 (74.1)		22 401 (68.1)	819 311 (74.2)	
Smoking							
Never	789 822 (69.4)	10 766 (70.0)	779 056 (69.4)	31.9^b^	23 487 (71.4)	766 335 (69.4)	88.5^b^
Former	198 707 (17.4)	2448 (15.9)	196 259 (17.5)		5108 (15.5)	193 599 (17.5)	
Active	149 001 (13.1)	2166 (14.1)	146 835 (13.1)		4315 (13.1)	144 686 (13.1)	
Alcohol consumption							
<7 Units/wk	960 329 (84.4)	13 285 (86.4)	947 044 (84.4)	65.5^b^	28 372 (86.2)	931 957 (84.4)	106.7^b^
≥7 Units/wk	177 201 (15.6)	2095 (13.6)	175 106 (15.6)		4538 (13.8)	172 663 (15.6)	
Clinical factors							
BMI							
<18.5	22 848 (2.0)	406 (2.6)	22 442 (2.0)	173.4^b^	755 (2.3)	22 093 (2.0)	681.5^b^
18.5-29.0	1 070 667 (94.1)	14 100 (91.7)	1 056 567 (94.2)		29 992 (91.1)	1 040 675 (94.2)	
≥30.0	44 015 (3.9)	874 (5.7)	43 141 (3.8)		2163 (6.6)	41 852 (3.8)	
BP, mm Hg							
SBP <90 or DBP <60	18 551 (1.6)	287 (1.9)	18 264 (1.6)	49.0^b^	613 (1.9)	17 938 (1.6)	102.6^b^
SBP ≤90 to <140; DBP ≤60 to <90 mm Hg	835 966 (73.5)	10 940 (71.1)	825 026 (73.5)		23 406 (71.1)	812 560 (73.6)	
SBP ≥140 or DBP ≥90	283 013 (24.9)	4153 (27.0)	278 860 (24.9)		8891 (27.0)	274 122 (24.8)	
FBG, mg/dL							
<126	996 820 (87.6)	13 180 (85.7)	983 640 (87.7)	51.9^b^	27 797 (84.5)	969 023 (87.7)	315.2^b^
≥126	140 710 (12.4)	2200 (14.3)	138 510 (12.3)		5113 (15.5)	135 597 (12.3)	
Hemoglobin, g/dL							
Men <14, women <12	264 961 (23.3)	4076 (26.5)	260 885 (23.3)	90.4^b^	7761 (23.6)	257 200 (23.3)	1.6
Men ≥14, women ≥12	872 569 (76.7)	11 304 (73.5)	861 265 (76.8)		25 149 (76.4)	847 420 (76.7)	
HDL-C, mg/dL							
<40	147 675 (13.0)	2401 (15.6)	145 274 (12.9)	98.3^b^	4847 (14.7)	142 828 (12.9)	90.9^b^
≥40	989 855 (87.0)	12 979 (84.4)	976 876 (87.1)		28 063 (85.3)	961 792 (87.1)	
LDL-C, mg/dL							
≥160	124 374 (10.9)	1737 (11.3)	122 637 (10.9)	2.3	3624 (11.0)	120 750 (10.9)	0.0
<160	1 013 156 (89.1)	13 643 (88.7)	999 513 (89.1)		29 286 (89.0)	983 870 (89.1)	
Triglycerides, mg/dL							
≥200	174 832 (15.4)	2604 (16.9)	172 228 (15.3)	29.6^b^	5513 (16.8)	169 319 (15.3)	48.7^b^
<200	962 698 (84.6)	12 776 (83.1)	949 922 (84.7)		27 397 (83.2)	935 301 (84.7)	
Hearing							
Intact	1 034 766 (91.0)	13 504 (87.8)	1 021 262 (91.0)	215.9^b^	28 307 (86.0)	1 006 459 (91.1)	1148.7^b^
Impaired (1 ear)	64 809 (5.7)	1094 (7.1)	63 715 (5.7)		2620 (8.0)	62 189 (5.6)	
Impaired (both ears)	37 955 (3.3)	782 (5.1)	37 173 (3.3)		1983 (6.0)	35 972 (3.3)	
Depression, mean (SD)	0.34 (0.76)	0.94 (1.15)	0.33 (0.77)	98.0^b^	0.90 (1.13)	0.32 (0.76)	135.0^b^
Medical comorbidities							
Hypertension							
Yes	447 643 (39.3)	6475 (42.1)	441 168 (39.3)	54.8^b^	14 341 (43.6)	433 302 (39.2)	264.2^b^
No	689 887 (60.6)	8905 (57.9)	680 982 (60.7)		18 569 (56.4)	671 318 (60.8)	
Diabetes							
Yes	162 717 (14.3)	2910 (18.9)	159 807 (14.2)	280.3^b^	6495 (19.7)	156 222 (14.1)	826.8^b^
No	974 813 (85.7)	12 470 (81.1)	962 343 (85.8)		26 415 (80.3)	948 398 (85.9)	
Dyslipidemia							
Yes	76 020 (6.7)	1059 (6.9)	74 961 (6.7)	0.6	2299 (7.0)	73 721 (6.7)	4.9^c^
No	1 061 510 (93.3)	14 321 (93.1)	1 047 189 (93.3)		30 611 (93.0)	1 030 899 (93.3)	
Heart disease							
Yes	58 547 (5.1)	1077 (7.0)	57 470 (5.1)	116.4^b^	2389 (7.3)	56 158 (5.1)	313.7^b^
No	1 078 983 (94.9)	14 303 (93.0)	1 064 680 (94.9)		30 521 (92.7)	1 048 462 (94.9)	
Stroke							
Yes	19 987 (1.8)	1168 (7.6)	18 819 (1.7)	3315.4^b^	1957 (5.9)	18 030 (1.6)	3577.3^b^
No	1 117 543 (98.2)	14 212 (92.4)	1 103 331 (98.3)		30 953 (94.1)	1 086 590 (98.4)	

Abbreviations: BMI, body mass index (calculated as weight in kilograms divided by height in meters squared); BP, blood pressure; DBP, diastolic BP; FBG, fasting blood glucose; HDL-C, high-density lipoprotein cholesterol; LDL-C, low-density lipoprotein cholesterol; MCR, motoric cognitive risk; OLS, one-leg-standing; SBP, systolic BP; TUG, timed-up-and-go.

SI conversion factors: To convert hemoglobin to grams per liter, multiply by 10.0; HDL-C to millimoles per liter, multiply by 0.0259; LDL-C to millimoles per liter, multiply by 0.0259; and triglycerides to millimoles per liter, multiply by 0.0113.

^a^ Differences were determined using χ^2^ or *t* tests.

^b^ *P* < .001 with multiple comparisons.

^c^ *P* < .05 with multiple comparisons.

A total of 15 380 individuals (1.4%) met the criteria for MCR-TUG and 32 910 (2.9%) participants met the criteria for MCR-OLS. The MCR and non-MCR groups differed significantly in most categories. The MCR group consisted of more women and Medicaid beneficiaries; exercised less regularly; smoked or consumed alcohol; and showed a tendency toward excessive body mass index, blood pressure, and fasting blood glucose and triglyceride levels. Furthermore, the MCR groups had higher prevalence of hearing impairment, depressive symptoms, and medical comorbidities of hypertension, diabetes, heart disease, and stroke.

After propensity score matching, 15 375 pairs were matched in the MCR-TUG group and 32 898 pairs were matched in the MCR-OLS groups, and the number of pairs matched in the impaired TUG, impaired OLS, and SCD groups are presented in the eFigure in Supplement 1. The absolute mean standardized differences of all covariates between the MCR and non-MCR groups (eTable 2 and eTable 3 in Supplement 1), and between the impaired TUG, impaired OLS, and SCD groups and their respective reference groups, were reduced to less than 0.1 after matching.

### Risk of Incident Dementia

The mean (SD) follow-up period was 7.02 (1.38) years, during which 55 760 incident dementia cases (4.9%) occurred. The MCR group exhibited a higher cumulative incidence of dementia (Figure) and significantly shorter follow-up period than those in non-MCR groups (MCR-TUG vs non–MCR-TUG: *t* = 18.73; *P* < .001; MCR-OLS vs non–MCR-OLS: *t* = 25.56; *P* < .001). However, follow-up periods were not significantly different (MCR-TUG vs MCR-OLS: *t* = 1.42; *P* = .15) between MCR subtypes. The dementia incidence rate in the MCR-TUG group was 18.55 per 1000 person-years and in the MCR-OLS group, 17.77 per 1000 person-years; the rate in the non–MCR-TUG group was 6.82 per 1000 person-years and in the non–MCR-OLS, 6.66 per 1000 person-years (Table 2).

**Figure.**
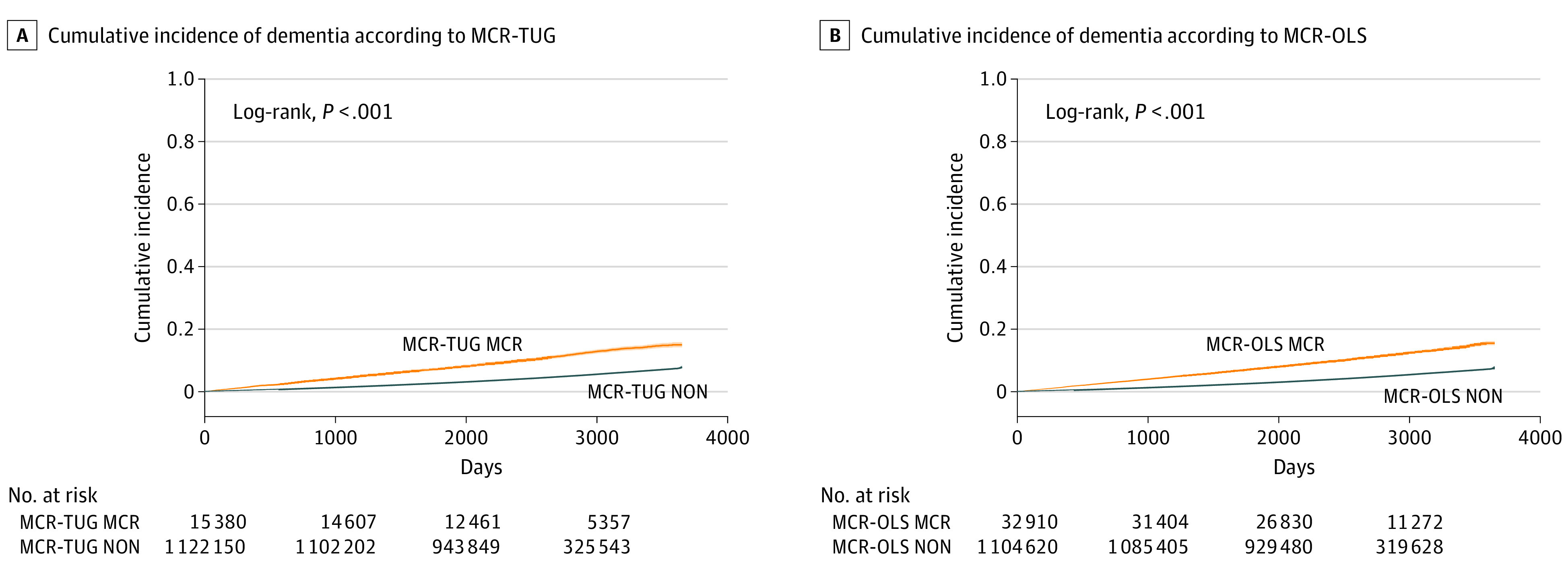
Cumulative Incidence of Dementia According to Motoric Cognitive Risk (MCR)–Timed-Up-and-Go (TUG) and MCR–One-Leg Standing (OLS) Tests The cumulative incidence of dementia is significantly higher in the MCR groups than in the non-MCR groups. NON indicates non-MCR group.

**Table 2.** Risk of Incident Dementia According to Impaired TUG, Impaired OLS, SCD, and MCR

Group	No. (%)		Events per 1000 person-years	AHR (95% CI)^a^
	Participants (n = 1 137 530)	Dementia events (n = 55 760)		
TUG				
Intact	1 050 666 (92.4)	49 245 (4.7)	6.68	1 [Reference]
Impaired	86 864 (7.6)	6515 (7.5)	10.68	1.43 (1.39-1.47)
OLS				
Intact	956 186 (84.1)	42 449 (4.4)	6.31	1 [Reference]
Impaired	181 344 (15.9)	13 311 (7.3)	10.56	1.49 (1.46-1.52)
SCD				
No	975 765 (85.8)	41 584 (4.3)	6.08	1 [Reference]
Yes	161 765 (14.2)	14 176 (8.8)	12.37	1.75 (1.72-1.79)
MCR-TUG				
Non-MCR	1 122 150 (98.6)	53 790 (4.8)	6.82	1 [Reference]
MCR	15 380 (1.4)	1970 (12.8)	18.55	2.03 (1.94-2.13)
MCR-OLS				
Non-MCR	1 104 620 (97.1)	51 707 (4.7)	6.66	1 [Reference]
MCR	32 910 (2.9)	4053 (12.3)	17.77	2.05 (1.98-2.12)

Abbreviations: AHR, adjusted hazard ratio; MCR, motoric cognitive risk; OLS, one-leg-standing; SCD, subjective cognitive decline; TUG, timed-up-and-go.

^a^ Adjusted for sex, income, lifestyle factors, clinical factors, and medical comorbidities (hypertension, diabetes, dyslipidemia, heart disease, and stroke).

On controlling all covariates, the dementia risk increased in the MCR groups (MCR-TUG: AHR, 2.03; 95% CI, 1.94-2.13; MCR-OLS: AHR, 2.05; 95% CI, 1.98-2.12). Impaired TUG, impaired OLS, and SCD were also associated with increased risk of incident dementia while controlling for all covariates (Table 2). In models 1 to 4, the AHR of dementia ranged from 2.03 to 2.65 in the MCR-TUG group and from 2.05 to 2.60 in the MCR-OLS group (eTable 4 in Supplement 1). Propensity score matching further supported these results (eTable 5 in Supplement 1).

### Risk of AD and VD

Among the 55 760 all-cause incident dementia events, 38 796 (69.6%) were observed in participants with AD and 6439 (11.5%) in those with VD. The MCR-TUG and MCR-OLS groups exhibited increased AD and VD risk (Table 3). Impaired TUG, impaired OLS, and SCD were also associated with an increased risk of AD and VD. The analysis of propensity score–matched data supported these outcomes (eTable 5 in Supplement 1).

**Table 3.** Risk of Alzheimer Disease and Vascular Dementia According to Impaired TUG, Impaired OLS, SCD, and MCR

Group	Events, No.	HR (95% CI)				
		Impaired TUG	Impaired OLS	SCD	MCR-TUG	MCR-OLS
Dementia (all-cause)	55 760	1.58 (1.54-1.62)	1.66 (1.63-1.70)	1.99 (1.95-2.03)	2.65 (2.54-2.77)	2.60 (2.52-2.69)
Alzheimer disease^a^	38 796	1.58 (1.53-1.63)	1.66 (1.62-1.70)	1.99 (1.95-2.04)	2.61 (2.47-2.75)	2.57 (2.48-2.67)
Vascular dementia^b^	6439	1.80 (1.68-1.94)	1.97 (1.87-2.09)	1.92 (1.82-2.03)	2.86 (2.52-3.24)	2.73 (2.49-2.99)

Abbreviations: HR, hazard ratio; MCR, motoric cognitive risk; OLS, one-leg-standing; SCD, subjective cognitive decline; TUG, timed-up-and-go.

^a^ Prescription of donepezil, galantamine, rivastigmine, or memantine with *International Statistical Classification of Diseases and Related Health Problems, Tenth Revision* (*ICD-10*) codes F00 or G30.

^b^ Prescription of donepezil, galantamine, rivastigmine, or memantine with *ICD-10* code F01.

### Sensitivity Analysis of MCR and Incident Dementia

After excluding 8526 participants (0.7%) diagnosed with dementia within 1 year of the index date (sensitivity analysis 1) and 141 818 outliers (12.4%) in the TUG and OLS (sensitivity analysis 2) groups (eFigure in Supplement 1), both MCR-TUG and MCR-OLS were consistently associated with increased risk of incident dementia (eTable 6 in Supplement 1). Impaired TUG, impaired OLS, and SCD were associated with the risk of incident dementia. The association of each with dementia, AD, and VD was consistent, despite subgroup exclusion. The analysis of propensity score–matched data supported the results of the sensitivity analysis (eTable 5 in Supplement 1).

## Discussion

In this study, an association between modified MCR and incident dementia observed was consistent with previous studies examining the estimation validity of MCR for dementia despite the heterogeneity of the study population, follow-up time, and MCR definition and dementia diagnosis among studies.^24,25^ A study of 26 802 participants across 22 cohorts in 17 countries reported a pooled MCR prevalence of approximately 10%,^24^ whereas the prevalence in our study was approximately 1% to 2%. This discrepancy may be attributed to the lower age and potentially higher cognitive health among participants in our study than in the multicohort study. Notably, MCR estimated dementia even in the mid-60s population.

The AHR for all-cause dementia of the modified MCR (MCR-TUG, 2.03; MCR-OLS, 2.05) was comparable with the AHR in the multicohort study (1.93),^24^ and MCR had incremental estimation validity of the cognitive (SCD) and motoric (TUG or OLS) components for dementia.^6,24^ The SCD was measured using the KDSQ-P, which showed similar results to examiner-reported assessment tools, such as the 15-item Consortium to Establish a Registry for Alzheimer’s Disease questionnaire^26^ or the Short Portable Mental Status Questionnaire.^27^ These results suggest that modified MCR may be used as a feasible screening method for dementia during national health examinations of the mid-60s population.^25^

However, a greater risk of incident dementia might have been observed if slow gait speed had been used to define MCR. An MCR subtype defined using the 5-times-sit-to-stand, which has a balance component, could not estimate cognitive decline as effectively as MCR defined with slow gait in a previous report.^28^ Since the choice of gait or balance task can influence outcomes, further research is needed regarding gait and balance as motoric parameters for identifying individuals at risk of dementia.

To our knowledge, this study is the first to use OLS instead of gait variables to define MCR. This novel MCR-OLS subtype was found to estimate the risk of dementia, similar to MCR-TUG. The OLS test requires postural balance maintenance through coordination between proprioception and the somatosensory system.^29^ Poor balance has been associated with parietal lobe and hippocampal dysfunction, which are related to visuospatial orientation and cognition.^13^ A previous study involving 686 community-dwelling individuals with AD suggested OLS as a marker of advanced dementia that estimated a higher rate of cognitive decline.^13^ Unlike gait variables, which require at least 3 m of straight aisle without obstacles, OLS measurement only requires sufficient space for people to stand with their arms spread; hence, implementation in rural or contactless health care settings is possible, making screening for at-risk status easier. Nonetheless, further research is needed to fully investigate the potential of OLS as a component of MCR to identify individuals at risk of dementia.

Our study found that both modified MCR subtypes were associated with a higher risk of developing AD and VD. A cohort study of 997 community-dwelling individuals in the US reported that MCR estimated the risk of VD but not AD,^6^ while a French study of 651 women reported an association between MCR and the incidence of all-cause dementia, including AD, but not non-AD dementia.^30^ A meta-analysis investigating the association between MCR and brain structures reported that MCR was associated with reduced gray matter volume in the prefrontal and premotor cortices, but not white matter abnormalities.^31^ These results suggest that neurodegenerative, rather than ischemic, lesions are primarily involved in MCR pathologic changes. When investigating the association between MCR and dementia incidence, adjusting for cardiovascular risk factors is important owing to their association with both MCR and dementia.^25^ We found that cardiovascular risk factors, namely, diabetes, hypertension, stroke, heart disease, smoking, and obesity, were significantly more prevalent in the MCR group than in the non-MCR group. We also observed a significant difference in AD incidence between the MCR and control groups after propensity score matching for known risk factors, suggesting that the estimation validity of MCR for dementia is not solely attributed to vascular abnormalities but also to neurodegeneration through other mechanisms, consistent with a previous neuroimaging study.^7^ Given that our study findings suggest that modified MCR is associated with both AD and VD, we advise that the MCR group participants be screened regularly for cognitive impairment; multidomain interventions, including nutrition, exercise, and cognitive training, be implemented to enhance cognitive reserve; and cardiovascular risk factors be reduced to prevent and/or delay the onset of dementia.^32,33^

The present study supports the validity of modified MCR subtypes as an estimator of dementia in a nationally representative homogeneous population after eliminating age-related confounding factors. Since neurophysiologic tests are often expensive and require professionals with specialized training for administration, MCR can be a simple and efficient alternative for identifying individuals at high risk for dementia. Moreover, MCR assessment is not influenced by educational level or learning effects following repeated testing, further enhancing its credibility and validity.

### Limitations

This study had several limitations. First, given the absence of objective neuropsychological tests, some patients may have been overlooked or misdiagnosed. As this study analyzed retrospective data, we relied on *ICD-10* codes and dementia medication prescriptions to infer the incidence of dementia. However, misclassification in the present study may have caused null hypothesis bias, resulting in underestimated outcomes.^34^ Sensitivity analysis that excluded participants diagnosed with dementia within 1 year of the index date helped to enhance the robustness of our findings. Second, controlling for other confounding variables that may impact dementia development, such as the *APOE* genotype, educational level, or imaging biomarkers, was not possible.^35,36^ Third, as all participants were aged 66 years, our findings may not comprehensively represent the older population; hence, generalizability may be limited.

## Conclusions

In this large nationwide cohort study of participants of the NSPTA aged 66 years, we found that modified MCR had higher AHRs of incident dementia, including AD and VD, than the individual cognitive or motoric components. Our findings suggest that modified MCR can be implemented as a practical screening tool for estimating dementia during national health examinations among individuals in their mid-60s. Further research is needed on the clinical and neurobiological aspects of MCR as a potential risk factor for dementia and the potential of MCR subtypes in identifying at-risk individuals.

## Notes

Supplement 1. **eMethods.** Detailed Methods **eTable 1.** Sex- and Test Condition–Specific Mean, Standard Deviation, and Cutoff Values of TUG and OLS Test **eTable 2.** Descriptive Characteristics and Standardized Mean Differences for the Original and Propensity Score–Matching Cohorts: MCR-TUG **eTable 3.** Descriptive Characteristics and Standardized Mean Differences for the Original and Propensity Score–Matching Cohorts: MCR-OLS **eTable 4.** Risk of Incident Dementia According to Impaired TUG, Impaired OLS, SCD, and MCR **eTable 5.** Propensity Score Matching Data Analysis: Risk of Incident Dementia According to Impaired TUG, Impaired OLS, SCD, and MCR **eTable 6.** Sensitivity Analysis for the Risk of Incident Dementia According to Impaired TUG, Impaired OLS, SCD, and MCR **eFigure.** A Flowchart of the Study Population

Supplement 2.
Data Sharing Statement
