# Supplementary material for: Motoric Cognitive Risk and Incident Dementia in Older Adults
Source: JAMA Netw Open. 2023 Oct 19;6(10):e2338534. doi: 10.1001/jamanetworkopen.2023.38534 (PMC10587785; doi:10.1001/jamanetworkopen.2023.38534)
Supplement: Supplement 1. — eMethods. Detailed Methods eTable 1. Sex- and Test Condition–Specific Mean, Standard Deviation, and Cutoff Values of TUG and OLS Test eTable 2. Descriptive Characteristics and Standardized Mean Differences for the Original and Propensity Score–Matching Cohorts: MCR-TUG eTable 3. Descriptive Characteristics and Standardized Mean Differences for the Original and Propensity Score–Matching Cohorts: MCR-OLS eTable 4. Risk of Incident Dementia According to Impaired TUG, Impaired OLS, SCD, and MCR eTable 5. Propensity Score Matching Data Analysis: Risk of Incident Dementia According to Impaired TUG, Impaired OLS, SCD, and MCR eTable 6. Sensitivity Analysis for the Risk of Incident Dementia According to Impaired TUG, Impaired OLS, SCD, and MCR eFigure. A Flowchart of the Study Population [file jamanetwopen-e2338534-s001.pdf]

Supplementary Online Content

Chung J, Byun S. Motoric cognitive risk and incident dementia in older adults. *JAMA Netw Open*. 2023;6(10):e2338534. doi:10.1001/jamanetworkopen.2023.38534

eMethods. Detailed Methods

eTable 1. Sex- and Test Condition–Specific Mean, Standard Deviation, and Cutoff Values of TUG and OLS Test

eTable 2. Descriptive Characteristics and Standardized Mean Differences for the Original and Propensity Score–Matching Cohorts: MCR-TUG

eTable 3. Descriptive Characteristics and Standardized Mean Differences for the Original and Propensity Score–Matching Cohorts: MCR-OLS

eTable 4. Risk of Incident Dementia According to Impaired TUG, Impaired OLS, SCD, and MCR

eTable 5. Propensity Score Matching Data Analysis: Risk of Incident Dementia According to Impaired TUG, Impaired OLS, SCD, and MCR

eTable 6. Sensitivity Analysis for the Risk of Incident Dementia According to Impaired TUG, Impaired OLS, SCD, and MCR

eFigure. A Flowchart of the Study Population

This supplementary material has been provided by the authors to give readers additional information about their work.

© 2023 Chung J et al. *JAMA Network Open.*

eMethods. Detailed Methods

Data sources

Data were obtained retrospectively from the Korean National Health Insurance (KNHI) database. As enrollment in the KNHI is mandatory in Korea, approximately 97% of the Korean population are subscribed, and the remaining 3% of individuals are covered through Medicaid.[1] Through the National Health Screening Program, Koreans receive regular health check-up from birth and every two years from the age of 20. In 2007, the National Screening Program for Transitional Ages (NSPTA) was launched as part of the national health examination to focus on pivotal life transition periods at the ages of 40 and 66.[2] The health screening performed in individuals aged 66 years includes additional components, such as physical and cognitive function assessment and osteoporosis screening beyond the standard checks.[2]

Since the KNHI acts as the single insurer in Korea and is responsible for all administrative processes and reimbursements related to medical service claims, the KNHI database contains health information and examination results for the entire population.[3] While collected for administrative purposes, the KNHI supports policy and academic research utilizing this information. The provision of this data requires official review and approval of research proposals, and Institutional Review Board (IRB) approval or exemption is mandatory. Since KNHI data provides only anonymized and de-identified information, the requirement for informed consent was waived.[1] Raw data cannot be exported and analysis is permitted only within specified rooms. In this study, the dataset was generated with the permission of the National Health Insurance Sharing Service (NHIS-2021-1-449).

Subjective cognitive decline (SCD) assessment

The pre-screening Korean Dementia Screening Questionnaire (KDSQ-P)[4] was implemented as part of the NSPTA as a preliminary survey for the KDSQ suitable for use exclusively in large group settings. The KDSQ-P is a self-reported questionnaire consisting of the following five questions: (1) Do you perceive your memory to be worse than that of your friends and colleagues?; (2) Do you believe your memory has regressed compared to its state a year ago?; (3) Are there any instances where your memory poses challenges during the execution of important tasks?; (4) Are individuals around you aware of the decline in your memory?; and (5) Do you consider yourself less adept at performing daily tasks that you were once proficient at? Each question permits self-assessment through three potential responses: "no," "occasionally yes," or "frequently yes," corresponding to scores of 0, 1, and 2, respectively. The cumulative scores of KDSQ-P range from 0 to 10, with higher scores indicating increased levels of SCD. Participants who scored ≥4 were advised to seek further evaluation.[4] In this study, participants who scored ≥4 from the KDSQ-P were classified as the SCD group.

© 2023 Chung J et al. *JAMA Network Open.*

eTable 1. Sex- and test condition-specific mean, standard deviation, and cut-off values of TUG and OLS test

	**TUG**		**OLS**			
Sex	Male	Female	Male		Female	
Test condition	-	-	Eyes opened	Eyes closed	Eyes opened	Eyes closed
N	526,902	610,628	414,651	112,251	483,822	126,806
M±SD (s)	8.3±3.4	8.7±3.4	20.3±9.0	13.0±8.6	18.0±8.6	11.3±8.0
Cut-off (s)*	≥12	≥13	≤11	≤4	≤9	≤3

* Time (s) of the original data were reported in whole numbers.
Abbreviations: TUG, timed-up-and-go; OLS, one-leg-standing; M, mean; SD, standard deviation

© 2023 Chung J et al. *JAMA Network Open.*

eTable 2. Descriptive characteristics and standardized mean differences for the original and propensity score–matching cohorts: MCR-TUG

	Participants, No. (%)					
	Original			PSM		
	MCR-TUG (n=15,380)	Non-MCR (n=1,122,150)	SMD	MCR-TUG (n=15,375)	Non-MCR (n=15,375)	SMD
Sex						
Male	6,268 (40.8)	520,634 (46.4)	.114	6,267 (40.8)	6,173 (40.1)	.012
Female	9,112 (59.2)	601,516 (53.6)		9,108 (59.2)	9,202 (59.9)	
Income						
1st to 8th	3,768 (24.5)	316,334 (28.2)	.198	3,763 (24.5)	3,755 (24.4)	.001
9th to 15th	4,989 (32.4)	362,092 (32.3)		4,989 (32.5)	4,995 (32.5)	
16th to 20th	5,245 (34.1)	407,677 (36.3)		5,245 (34.1)	5,238 (34.1)	
Medicaid	1,378 (9.0)	36,047 (3.2)		1,378 (9.0)	1,387 (9.0)	
**Lifestyle factors**						
Physical activity						
Never	5,112 (33.2)	290,706 (25.9)	.161	5,107 (33.2)	5,117 (33.3)	.001
≥Once a week	10,268 (66.8)	831,444 (74.1)		10,268 (66.8)	10,258 (66.7)	
Smoking						
Never	10,766 (70.0)	779,056 (69.4)	.048	10,762 (70.0)	10,866 (70.7)	.015
Quit	2,448 (15.9)	196,259 (17.5)		2,447 (15.9)	2,376 (15.5)	
Active	2,166 (14.1)	146,835 (13.1)		2,166 (14.1)	2,133 (13.9)	
Alcohol consumption						
<7 units/week	13,285 (86.4)	947,044 (84.4)	.056	13,280 (86.4)	13,288 (86.4)	.002
≥7 units/week	2,095 (13.6)	175,106 (15.6)		2,095 (13.6)	2,087 (13.6)	
**Clinical factors**						
BMI (kg/m^2)						
<18.5	406 (2.6)	22,442 (2.0)	.098	405 (2.6)	393 (2.6)	.006
18.5~30	14,100 (91.7)	1,056,567 (94.2)		14,097 (91.7)	14,119 (91.8)	
≥30	874 (5.7)	43,141 (3.8)		873 (5.7)	863 (5.6)	
BP (mmHg)						
SBP<90 or DBP<60	287 (1.9)	18,264 (1.6)	.054	287 (1.9)	262 (1.7)	.012
90≤SBP<140 60≤DBP<90	10,940 (71.1)	825,026 (73.5)		10,938 (71.1)	10,949 (71.2)	
SBP≥140 or DBP≥90	4,153 (27.0)	278,860 (24.9)		4,150 (27.0)	4,164 (27.1)	
FBS (mg/dL)						
<126	13,180 (85.7)	983,640 (87.7)	.058	13,178 (85.7)	13,207 (85.9)	.005
≥126	2,200 (14.3)	138,510 (12.3)		2,197 (14.3)	2,168 (14.1)	
Hemoglobin (g/dL)						
M<14, F<12	4,076 (26.5)	260,885 (23.3)	.058	4,066 (26.4)	3,999 (26.0)	.011
M≥14, F≥12	11,304 (73.5)	861,265 (76.8)		11,309 (73.6)	11,376 (74.0)	

© 2023 Chung J et al. *JAMA Network Open.*

	Participants, No. (%)					
	Original			PSM		
	MCR-TUG (n=15,380)	Non-MCR (n=1,122,150)	SMD	MCR-TUG (n=15,375)	Non-MCR (n=15,375)	SMD
HDL (mg/dL)						
<40	2,401 (15.6)	145,274 (12.9)	.076	2,399 (15.6)	2,312 (15.0)	.016
≥40	12,979 (84.4)	976,876 (87.1)		12,976 (84.4)	13,063 (85.0)	
LDL (mg/dL)						
≥160	1,737 (11.3)	122,637 (10.9)	.012	1,737 (11.3)	1,708 (11.1)	.006
<160	13,643 (88.7)	999,513 (89.1)		13,638 (88.7)	13,667 (88.9)	
Triglyceride (mg/dL)						
≥200	2,604 (16.9)	172,228 (15.3)	.043	2,603 (16.9)	2,547 (16.6)	.010
<200	12,776 (83.1)	949,922 (84.7)		12,772 (83.1)	12,828 (83.4)	
Hearing						
Intact	13,504 (87.8)	1,021,262 (91.0)	.109	13,503 (87.8)	13,548 (88.1)	.011
Impaired (One ear)	1,094 (7.1)	63,715 (5.7)		1,094 (7.1)	1,051 (6.8)	
Impaired (Both ears)	782 (5.1)	37,173 (3.3)		778 (5.1)	776 (5.0)	
Depression, mean (SD)	0.94 (1.15)	0.33 (0.77)	.624	0.94 (1.15)	0.94 (1.15)	.002
**Medical comorbidities**						
Hypertension						
Yes	6,475 (42.1)	441,168 (39.3)	.057	6,470 (42.1)	6,405 (41.7)	.009
No	8,905 (57.9)	680,982 (60.7)		8,905 (57.9)	8,970 (58.3)	
Diabetes mellitus						
Yes	2,910 (18.9)	159,807 (14.2)	.126	2,905 (18.9)	2,890 (18.8)	.002
No	12,470 (81.1)	962,343 (85.8)		12,470 (81.1)	12,485 (81.2)	
Dyslipidemia						
Yes	1,059 (6.9)	74,961 (6.7)	.008	1,059 (6.9)	989 (6.4)	.018
No	14,321 (93.1)	1,047,189 (93.3)		14,316 (93.1)	14,386 (93.6)	
Heart disease						
Yes	1,077 (7.0)	57,470 (5.1)	.079	1,075 (7.0)	1,059 (6.9)	.004
No	14,303 (93.0)	1,064,680 (94.9)		14,300 (93.0)	14,316 (93.1)	
Stroke						
Yes	1,168 (7.6)	18,819 (1.7)	.284	1,165 (7.6)	1,165 (7.6)	<.001
No	14,212 (92.4)	1,103,331 (98.3)		14,210 (92.4)	14,210 (92.4)	

Abbreviations: PSM, propensity score matching; SMD, standardized mean difference; MCR, motoric cognitive risk; TUG, timed-up-and-go; BMI, body mass index; BP, blood pressure; SBP, systolic blood pressure; DBP, diastolic blood pressure; FBS, fasting blood sugar; M, male; F, female; HDL, high-density lipoprotein; LDL, low-density lipoprotein; SD, standard deviation

SI conversion factors: To convert hemoglobin to g/L, multiply by 10.0; HDL to mmol/L, multiply by 0.0259; LDL to mmol/L, multiply by 0.0259; triglycerides to mmol/L, multiply by 0.0113.

© 2023 Chung J et al. *JAMA Network Open.*

eTable 3. Descriptive characteristics and standardized mean differences for the original and propensity score–matching cohorts: MCR-OLS

	Participants, No. (%)					
	Original			PSM		
	MCR-OLS (n=32,910)	Non-MCR (n=1,104,620)	SMD	MCR-OLS (n=32,898)	Non-MCR (n=32,898)	SMD
Sex						
Male	12,567 (38.2)	514,335 (46.6)	.170	12,563 (38.2)	12,346 (37.5)	.014
Female	20,343 (61.8)	590,285 (53.4)		20,335 (61.8)	20,552 (62.5)	
Income						
1st to 8th	8,060 (24.5)	312,042 (28.3)	.176	8,057 (24.5)	7,942 (24.1)	.025
9th to 15th	10,699 (32.5)	356,382 (32.3)		10,698 (32.5)	10,728 (32.6)	
16th to 20th	11,492 (34.9)	401,430 (36.3)		11,490 (34.9)	11,514 (35.0)	
Medicaid	2,659 (8.1)	34,766 (3.2)		2,653 (8.1)	2,714 (8.3)	
**Lifestyle factors**						
Physical activity						
Never	10,509 (31.9)	285,309 (25.8)	.135	10,504 (31.9)	10,416 (31.7)	.006
≥Once a week	22,401 (68.1)	819,311 (74.2)		22,394 (68.1)	22,482 (68.3)	
Smoking						
Never	23,487 (71.4)	766,335 (69.4)	.055	23,480 (71.4)	23,722 (72.1)	.017
Quit	5,108 (15.5)	193,599 (17.5)		5,104 (15.5)	4,927 (15.0)	
Active	4,315 (13.1)	144,686 (13.1)		4,314 (13.1)	4,249 (12.9)	
Alcohol consumption						
<7 units/week	28,372 (86.2)	931,957 (84.4)	.052	28,361 (86.2)	28,439 (86.4)	.007
≥7 units/week	4,538 (13.8)	172,663 (15.6)		4,537 (13.8)	4,459 (13.6)	
**Clinical factors**						
BMI (kg/m^2)						
<18.5	755 (2.3)	22,093 (2.0)	.128	754 (2.3)	665 (2.0)	.019
18.5~30	29,992 (91.1)	1,040,675 (94.2)		29,989 (91.2)	30,091 (91.5)	
≥30	2,163 (6.6)	41,852 (3.8)		2,155 (6.6)	2,142 (6.5)	
BP (mmHg)						
SBP<90 or DBP<60	613 (1.9)	17,938 (1.6)	.055	612 (1.9)	532 (1.6)	.019
90≤SBP<140 60≤DBP<90	23,406 (71.1)	812,560 (73.6)		23,401 (71.1)	23,480 (71.4)	
SBP≥140 or DBP≥90	8,891 (27.0)	274,122 (24.8)		8,885 (27.0)	8,886 (27.0)	
FBS (mg/dL)						
<126	27,797 (84.5)	969,023 (87.7)	.094	27,790 (84.5)	27,900 (84.8)	.009
≥126	5,113 (15.5)	135,597 (12.3)		5,108 (15.5)	4,998 (15.2)	
Hemoglobin (g/dL)						
M<14, F<12	7,761 (23.6)	257,200 (23.3)	.135	7,757 (23.6)	7,489 (22.8)	.019

© 2023 Chung J et al. *JAMA Network Open.*

	Participants, No. (%)					
	Original			PSM		
	MCR-OLS (n=32,910)	Non-MCR (n=1,104,620)	SMD	MCR-OLS (n=32,898)	Non-MCR (n=32,898)	SMD
M≥14, F≥12	25,149 (76.4)	847,420 (76.7)		25,141 (76.4)	25,409 (77.2)	
HDL (mg/dL)						
<40	4,847 (14.7)	142,828 (12.9)	.052	4,845 (14.7)	4,695 (14.3)	.013
≥40	28,063 (85.3)	961,792 (87.1)		28,053 (85.3)	28,203 (85.7)	
LDL (mg/dL)						
≥160	3,624 (11.0)	120,750 (10.9)	.003	3,622 (11.0)	3,544 (10.8)	.008
<160	29,286 (89.0)	983,870 (89.1)		29,276 (89.0)	29,354 (89.2)	
Triglyceride (mg/dL)						
≥200	5,513 (16.8)	169,319 (15.3)	.039	5,510 (16.7)	5,333 (16.2)	.015
<200	27,397 (83.2)	935,301 (84.7)		27,388 (83.3)	27,565 (83.8)	
Hearing						
Intact	28,307 (86.0)	1,006,459 (91.1)	.166	28,305 (86.0)	28,415 (86.4)	.011
Impaired (One ear)	2,620 (8.0)	62,189 (5.6)		2,619 (8.0)	2,520 (7.7)	
Impaired (Both ears)	1,983 (6.0)	35,972 (3.3)		1,974 (6.0)	1,963 (6.0)	
Depression, mean (SD)	.90 (1.13)	.32 (.76)	.603	.90 (1.13)	.90 (1.12)	<.001
**Medical comorbidities**						
Hypertension						
Yes	14,341 (43.6)	433,302 (39.2)	.088	14,330 (43.6)	14,227 (43.2)	.006
No	18,569 (56.4)	671,318 (60.8)		18,568 (56.4)	18,671 (56.8)	
Diabetes mellitus						
Yes	6,495 (19.7)	156,222 (14.1)	.150	6,485 (19.7)	6,342 (19.3)	.011
No	26,415 (80.3)	948,398 (85.9)		26,413 (80.3)	26,556 (80.7)	
Dyslipidemia						
Yes	2,299 (7.0)	73,721 (6.7)	.012	2,298 (7.0)	2,153 (6.5)	.018
No	30,611 (93.0)	1,030,899 (93.3)		30,600 (93.0)	30,745 (93.5)	
Heart disease						
Yes	2,389 (7.3)	56,158 (5.1)	.090	2,384 (7.2)	2,279 (6.9)	.012
No	30,521 (92.7)	1,048,462 (94.9)		30,514 (92.8)	30,619 (93.1)	
Stroke						
Yes	1,957 (5.9)	18,030 (1.6)	.227	1,945 (5.9)	1,955 (5.9)	.001
No	30,953 (94.1)	1,086,590 (98.4)		30,953 (94.1)	30,943 (94.1)	

Abbreviations: PSM, propensity score matching; SMD, standardized mean difference, MCR, motoric cognitive risk; OLS, one-leg-standing; BMI, body mass index; BP, blood pressure; SBP, systolic blood pressure; DBP, diastolic blood pressure; FBS, fasting blood sugar; M, male; F, female; HDL, high-density lipoprotein; LDL, low-density lipoprotein; SD, standard deviation

SI conversion factors: To convert hemoglobin to g/L, multiply by 10.0; HDL to mmol/L, multiply by 0.0259; LDL to mmol/L, multiply by 0.0259; triglycerides to mmol/L, multiply by 0.0113.

© 2023 Chung J et al. *JAMA Network Open.*

eTable 4. Risk of incident dementia according to Impaired TUG, Impaired OLS, SCD, and MCR

	Impaired TUG (ref=Intact)	Impaired OLS (ref=Intact)	SCD (ref=Non-SCD)	MCR-TUG (ref=Non-MCR)	MCR-OLS (ref=Non-MCR)
Unadjusted HR (95% CI)	1.58 (1.54-1.62)	1.66 (1.63-1.70)	1.99 (1.95-2.03)	2.65 (2.54-2.77)	2.60 (2.52-2.69)
aHR in Model 1 (95% CI)[a]	1.58 (1.54-1.62)	1.64 (1.61-1.68)	1.94 (1.90-1.98)	2.59 (2.47-2.71)	2.52 (2.44-2.60)
aHR in Model 2 (95% CI)[b]	1.55 (1.51-1.59)	1.62 (1.59-1.65)	1.94 (1.90-1.98)	2.53 (2.42-2.65)	2.48 (2.40-2.56)
aHR in Model 3 (95% CI)[c]	1.48 (1.44-1.52)	1.54 (1.51-1.57)	1.79 (1.76-1.83)	2.19 (2.09-2.29)	2.17 (2.10-2.24)
aHR in Model 4 (95% CI)[d]	1.43 (1.39-1.47)	1.49 (1.46-1.52)	1.75 (1.72-1.79)	2.03 (1.94-2.13)	2.05 (1.98-2.12)

[a]Adjusted for sex and income
[b]Adjusted for sex, income, and lifestyle factors (physical activities, smoking, and alcohol consumption)
[c]Adjusted for sex, income, lifestyle factors, and clinical factors (BMI, BP, FBS, HDL, LDL, triglycerides, hearing, and depressive symptoms).
[d]Adjusted for sex, income, lifestyle factors, clinical factors, and medical comorbidities (hypertension, diabetes, dyslipidemia, heart disease, and stroke)

Abbreviations: TUG, timed-up-and-go; OLS, one-leg-standing; SCD, subjective cognitive decline; MCR, motoric cognitive risk; HR, hazard ratio; CI, confidence interval; aHR, adjusted hazard ratio; BMI, body mass index; BP, blood pressure; FBS, fasting blood sugar; HDL, high-density lipoprotein; LDL, low-density lipoprotein.

© 2023 Chung J et al. *JAMA Network Open.*

eTable 5. Propensity score matching data analysis: risk of incident dementia according to Impaired TUG, Impaired OLS, SCD, and MCR

Group	Impaired TUG		Impaired OLS		SCD		MCR-TUG		MCR-OLS	
	n (event)	HR (95% CI)	n (event)	HR (95% CI)	n (event)	HR (95% CI)	n (event)	HR (95% CI)	n (event)	HR (95% CI)
**Original population**										
Dementia (all-cause)	173,678 (11,324)	1.34 (1.29-1.39)	362,432 (22,793)	1.42 (1.39-1.46)	323,214 (22,134)	1.74 (1.69-1.79)	30,750 (2,979)	1.95 (1.81-2.11)	65,796 (6,035)	2.06 (1.95-2.17)
AD[a]	173,678 (7,873)	1.34 (1.28-1.40)	362,432 (15,794)	1.43 (1.39-1.48)	323,214 (15,466)	1.73 (1.68-1.79)	30,750 (2,093)	1.87 (1.71-2.05)	65,796 (4,205)	2.05 (1.92-2.19)
VD[b]	173,678 (1,452)	1.37 (1.23-1.52)	362,432 (2,910)	1.52 (1.41-1.63)	323,214 (2,519)	1.66 (1.53-1.80)	30,750 (375)	2.03 (1.63-2.52)	65,796 (760)	1.94 (1.67-2.26)
**Sensitivity analysis I[*]**										
Dementia (all-cause)	171,362 (10,462)	1.34 (1.29-1.39)	357,960 (21,235)	1.41 (1.37-1.45)	319,072 (20,438)	1.70 (1.65-1.74)	30,004 (2,693)	1.88 (1.74-2.04)	64,354 (5,502)	1.96 (1.85-2.07)
AD[a]	171,362 (7,263)	1.36 (1.30-1.42)	357,960 (14,834)	1.41 (1.36-1.45)	319,072 (14,334)	1.68 (1.63-1.74)	30,004 (1,882)	1.87 (1.70-2.05)	64,354 (3,842)	1.99 (1.86-2.13)
VD[b]	171,362 (1,325)	1.35 (1.21-1.51)	357,960 (2,615)	1.59 (1.47-1.72)	319,072 (2,302)	1.65 (1.52-1.80)	30,004 (323)	2.23 (1.76-2.83)	64,354 (700)	1.78 (1.52-2.08)
**Sensitivity analysis II[†]**										
Dementia (all-cause)	119,310 (7,241)	1.26 (1.21-1.32)	320,580 (19,337)	1.38 (1.34-1.42)	283,184 (19,604)	1.72 (1.67-1.77)	19,924 (1,798)	1.84 (1.67-2.03)	56,872 (4,967)	1.93 (1.82-2.04)
AD[a]	119,310 (5,019)	1.28 (1.21-1.35)	320,580 (13,432)	1.38 (1.33-1.43)	283,184 (13,291)	1.72 (1.66-1.79)	19,924 (1,224)	1.82 (1.62-2.04)	56,872 (3,494)	1.89 (1.76-2.02)
VD[b]	119,310 (861)	1.32 (1.15-1.51)	320,580 (2,366)	1.46 (1.34-1.58)	283,184 (2,124)	1.62 (1.48-1.77)	19,924 (216)	1.70 (1.29-2.24)	56,872 (566)	1.86 (1.56-2.21)

[*] Analysis excluding individuals diagnosed with dementia within one year of the index date
[†] Analysis excluding the outliers of TUG and OLS tests
[a] Prescription of donepezil, galantamine, rivastigmine, or memantine with ICD-10 code F00, G30
[b] Prescription of donepezil, galantamine, rivastigmine, or memantine with ICD-10 code F01

Abbreviations: PSM, propensity score matching; TUG, timed-up-and-go; OLS, one-leg-standing; SCD, subjective cognitive decline; MCR, motoric cognitive risk; HR, hazard ratio, CI, confidence interval; AD, Alzheimer’s disease; VD, vascular dementia

© 2023 Chung J et al. *JAMA Network Open.*

eTable 6. Sensitivity analysis for the risk of incident dementia according to Impaired TUG, Impaired OLS, SCD, and MCR

	Participants	Event	HR (95% CI)				
			Impaired TUG	Impaired OLS	SCD	MCR-TUG	MCR-OLS
**Original population**	1,137,530	55,760	1.58 (1.54-1.62)	1.66 (1.63-1.70)	1.99 (1.95-2.03)	2.65 (2.54-2.77)	2.60 (2.52-2.69)
**Sensitivity analysis I***							
Dementia (all-cause)	1,129,004	52,423	1.55 (1.51-1.59)	1.64 (1.61-1.68)	1.92 (1.88-1.96)	2.52 (2.41-2.65)	2.48 (2.40-2.57)
AD[a]		36,549	1.55 (1.50-1.60)	1.64 (1.60-1.68)	1.93 (1.89-1.97)	2.50 (2.36-2.64)	2.48 (2.38-2.58)
VD[b]		6,011	1.75 (1.62-1.89)	1.93 (1.82-2.04)	1.86 (1.76-1.98)	2.73 (2.39-3.12)	2.62 (2.38-2.88)
**Sensitivity analysis II†**							
Dementia (all-cause)	995,712	48,298	1.41 (1.37-1.46)	1.56 (1.53-1.59)	1.96 (1.92-2.00)	2.38 (2.25-2.53)	2.41 (2.33-2.50)
AD[a]		33,599	1.41 (1.36-1.47)	1.56 (1.52-1.60)	1.97 (1.92-2.02)	2.33 (2.18-2.50)	2.40 (2.30-2.50)
VD[b]		5,562	1.50 (1.36-1.64)	1.74 (1.64-1.85)	1.84 (1.73-1.96)	2.37 (2.00-2.81)	2.31 (2.08-2.56)

* Analysis excluding individuals diagnosed with dementia within one year of the index date
† Analysis excluding the outliers of TUG and OLS tests
[a] Prescription of donepezil, galantamine, rivastigmine, or memantine with ICD-10 code F00, G30
[b] Prescription of donepezil, galantamine, rivastigmine, or memantine with ICD-10 code F01

Abbreviations: TUG, timed-up-and-go; OLS, one-leg-standing; SCD, subjective cognitive decline; MCR, motoric cognitive risk; HR, hazard ratio; CI, confidence interval; AD, Alzheimer's disease; VD, vascular dementia

© 2023 Chung J et al. *JAMA Network Open.*

eFigure 1. A flowchart of the study population

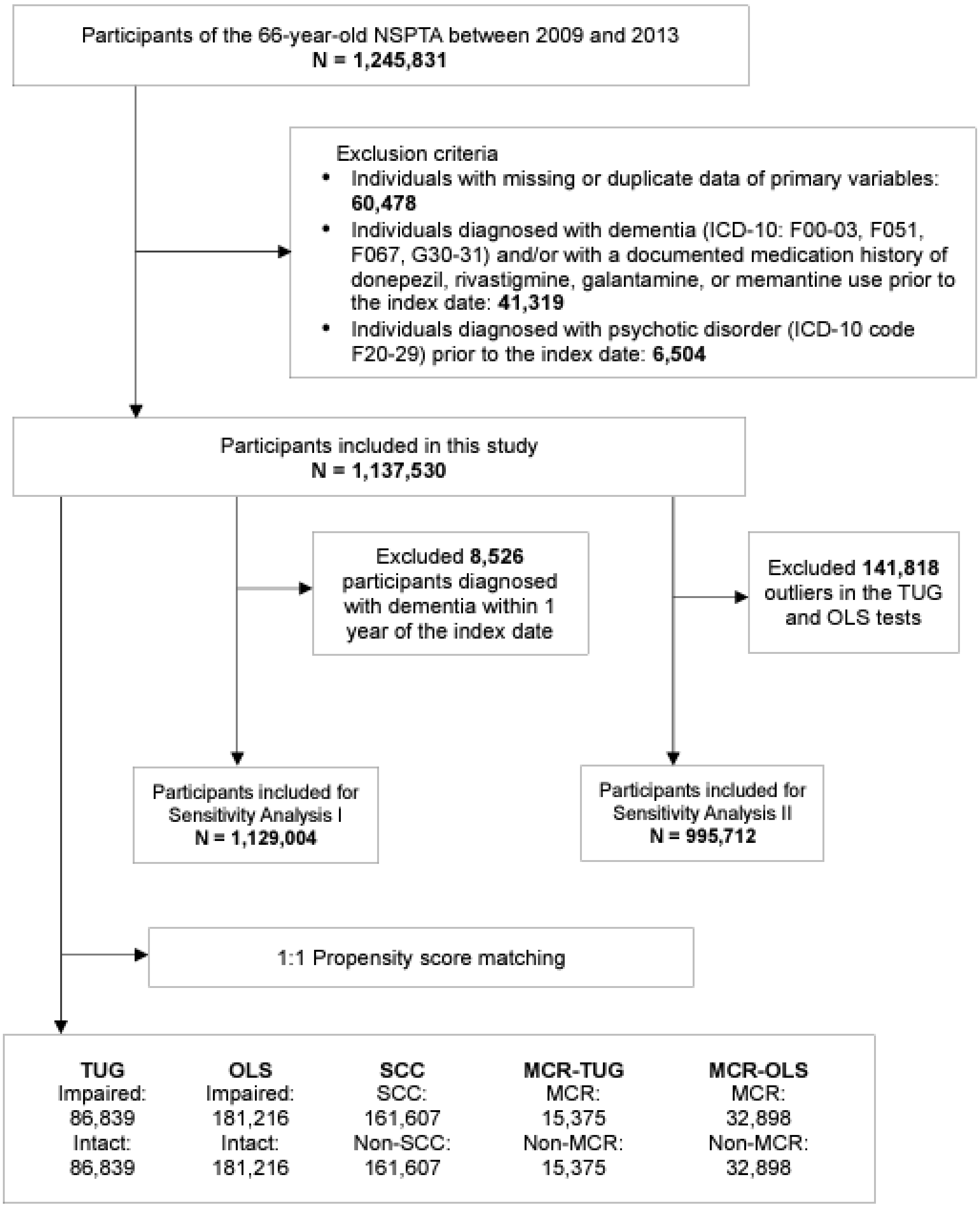

Abbreviations: NSPTA, National Screening Program for Transitional Ages; ICD-10, International Classification of Disease 10th revision; TUG, timed up and go; OLS, one-leg standing; MCR, motoric cognitive risk; SCC, subjective cognitive complaints

© 2023 Chung J et al. *JAMA Network Open.*

References

1. Shin DW, Cho J, Park JH, Cho B. National General Health Screening Program in Korea: history, current status, and future direction. *Precision and Future Medicine*. 2022;6(1):9-31. doi:10.23838/pfm.2021.00135

2. Kim HS, Shin DW, Lee WC, Kim YT, Cho B. National screening program for transitional ages in Korea: a new screening for strengthening primary prevention and follow-up care. *J Korean Med Sci*. 2012;27(Suppl):S70-S75. doi:10.3346/jkms.2012.27.S.S70

3. Cheol Seong S, Kim YY, Khang YH, et al. Data Resource Profile: The National Health Information Database of the National Health Insurance Service in South Korea. *Int J Epidemiol*. 2017;46(3):799-800. doi:10.1093/ije/dyw253

4. Jeon Y, Yun K, Kim Y. Validation of KDSQ-P as selecting elderly for KDSQ-C. *Korean J Health Promot*. 2010;10(2):45-52.

© 2023 Chung J et al. *JAMA Network Open.*
