# Supplementary material for: Motoric Cognitive Risk and Incident Dementia in Older Adults
Source: JAMA Netw Open. 2023 Oct 19;6(10):e2338534. doi: 10.1001/jamanetworkopen.2023.38534 (PMC10587785; doi:10.1001/jamanetworkopen.2023.38534)
Supplement: Supplement 2. — Data Sharing Statement [file jamanetwopen-e2338534-s002.pdf]

Data Sharing Statement

Chung. Motoric Cognitive Risk and Incident Dementia in Older Adults. *JAMA Netw Open*. Published October 19, 2023. doi:10.1001/jamanetworkopen.2023.38534

Data
Data available: No
